# Syphilis clustering among young pregnant women (18–35 years) in Kampala and Wakiso districts, Uganda

**DOI:** 10.3389/frph.2026.1829745

**Published:** 2026-07-15

**Authors:** Rogers Nsubuga, Timothy R. Muwonge, Andrew Mujugira, Barbara Castelnuvo, Edith Nakku-Joloba, Rosalind Parkes-Ratanshi, Yukari C. Manabe, Agnes N. Kiragga

**Affiliations:** 1 Research Department, Infectious Diseases Institute, Makerere University, Kampala, Uganda; 2 Department of Global Health, University of Washington, Seattle, WA, United States; 3 School of Public Health, Makerere University, Kampala, Uganda; 4 Centre for Public Health, Queens University, Belfast, United Kingdom; 5 Division of Infectious Diseases, Department of Medicine, Johns Hopkins University School of Medicine, Baltimore, MD, United States; 6 African Population and Health Research Centre, Nairobi, Kenya

**Keywords:** HIV, Morans I, spatial clustering, syphilis, Uganda, young women

## Abstract

**Introduction:**

In Uganda, spatial distribution of syphilis varies by age, gender, and region. Identifying clusters (subsets of administrative subdivisions) with high syphilis prevalence could boost efforts to eliminate mother-to-child transmission of syphilis. We examined spatial variations and clustering of syphilis prevalence among pregnant young women aged 18–35 years in Central Uganda.

**Methods:**

We analysed secondary data from a randomised trial that evaluated the effectiveness of three antenatal syphilis partner notification approaches (NCT02262390). This study analysed clustering of syphilis prevalence by administrative division in Kampala and Wakiso districts, using Moran's I tests and Local Indicator of Spatial Association (LISA). We used Kulldorff Spatial-Scan Poisson model to detect syphilis clustering and classified divisions into high- or low-prevalence (HP/LP) syphilis clusters based on 95% statistical significance. We estimated prevalence ratios for sociodemographic and bio-behavioural HIV risk factors associated with residence in HP divisions using modified Poisson regression.

**Results:**

Of 422 women diagnosed with syphilis, 26 (6%) had HIV and syphilis. Median age was 26 years (IQR 24–29). Most (314, 74%) were in monogamous marriages, and half (50%) had attained secondary education. Syphilis prevalence clustering was negatively associated with residence in HP divisions among women in polygamous marriages [adjusted prevalence ratio (APR) = 0.64; 95%: 0.47–0.88], those with an unplanned pregnancy (APR = 0.78; 95% CI: 0.64–0.93), and who had HIV testing >3 months prior (APR = 0.83, 95% CI: 0.72–0.95). Syphilis prevalence was significantly higher in 3/12 clusters: Kasangati Town Council [Relative Risk (RR) = 2.79, *p* < 0.0001], Kawempe (RR = 2.52, *p* < 0.0001), and Nabweru (RR = 1.95, *p* = 0.0002), and lower in one cluster–Kyengera Town Council (RR = 0.12, *p* < 0.0001). Notably, no significant clustering was detected among women with HIV (*p* > 0.05). Random patterns of syphilis prevalence were detected across all divisions (Moran's I = 0.08, *p* = 0.19). However, some neighbouring divisions had similar prevalence: Kawempe (1.06, *p* = 0.02) and Nabweru (0.54, *p* = 0.045). LISA analysis confirmed high syphilis prevalence in northern divisions (Kawempe and Nabweru; *p* = 0.01). By contrast, Central Region had neighbouring low and high prevalence divisions (Kawempe and Central; *p* = 0.001).

**Conclusion:**

Syphilis prevalence was similar within neighbouring divisions, but highest in Kasangati Town Council and Kawempe. Integrating spatial analysis in routine surveillance will enable detection of clusters where interventions can be targeted to eliminate congenital syphilis.

## Introduction

In sub-Saharan Africa (SSA), syphilis remains a significant public health challenge, especially among pregnant women (1). If untreated, the infections can result in adverse pregnancy outcomes, including congenital syphilis (2). Studies indicate higher STI prevalence, including syphilis, among younger women compared with older women (3, 4). In Uganda, syphilis prevalence was 1.3% among school-going girls (5), 3% among pregnant women attending facility antenatal clinics, higher in urban and peri-urban settings, and was more common among those with HIV (6.2%) than without (1.8%) (5, 6). While elimination of mother-to-child transmission of syphilis is a global health priority, entailing universal syphilis screening and treatment in pregnancy (7), the spatial distribution of syphilis cases within urban centres is not well characterized Thus, identifying high-prevalence clusters in which pregnant women have active syphilis infection is key to preventing adverse birth outcomes (8).

Understanding the geographic clustering of syphilis among pregnant women is critical for optimizing resource allocation and tailoring interventions to areas of greatest need. A scoping review demonstrated that mapping these patterns highlighted high-burden areas and helped uncover potential environmental and social determinants influencing disease transmission (9). Spatial analysis has been applied to detect localized HIV clusters in urban settings, revealing overlaps with other STIs and guiding targeted public health interventions (10). Such analyses can inform targeted public health strategies, including intensified screening, partner notification, and community engagement in high-burden areas, thereby supporting efforts to eliminate congenital syphilis. Spatial clustering techniques are essential for statistical considerations and form the initial steps in developing a model that predicts disease risk sites (11, 12). In this study, we examined the spatial distribution and clustering of syphilis prevalence among pregnant young women aged less than 35 years in the administrative divisions of Kampala and Wakiso districts in Central Uganda. By integrating spatial epidemiological methods with sociodemographic and behavioural data, we also identified divisions with high syphilis risk and investigated potential factors associated with syphilis clustering.

## Methods

### Study settings and design

This was a secondary analysis of data from the Syphilis Treatment of Partners (STOP) study. This randomised clinical trial compared the effectiveness of three partner notification strategies after antenatal syphilis screening among ∼17,000 pregnant women in Uganda (https://www.ClinicalTrials.gov, NCT02262390, 2014-October-08) (13). This parent trial took place from 12th February 2015 to 17th February 2016 among women attending antenatal care at Mulago Hospital.

Syphilis testing was conducted at enrolment using a treponemal point-of-care lateral flow assay (SD Bioline, Standard Diagnostics, Inc., Republic of Korea) and confirmed with non-treponemal testing using a lipoidal rapid plasma reagin (RPR) test (Fortress Diagnostics, United Kingdom). RPRs are not readily available in Uganda outside of studies and are not held in a syphilis registry to confirm treatment response or reinfection. Pregnant women and their male partners received free treatment for syphilis if they tested positive. HIV testing was performed using serial rapid diagnostic HIV tests in accordance with national guidelines*: Determine® HIV1/2 (Abbott, IL) [screening test], Stat-Pak HIV1/2 (Chembio Diagnostic Systems, Medford, NY) [confirmatory test], and SD-Bioline (Standard Diagnostics, Inc., Republic of Korea) [tie breaker test]* (14).

### Study population

For this analysis, we included women aged between 18 and 35 years, who had a positive syphilis test at parent trial enrolment and resided within one of the 12 administrative divisions, including Kampala city divisions and neighbouring Wakiso district town councils. Women older than 35 years or residing outside the study catchment area were excluded. Additionally, we considered sociodemographic and behavioural data collected at enrolment, including age, marital status, education, employment, pregnancy history, and HIV testing history. HIV testing history (>3 months prior) was self-reported and reflected previous testing behaviour but did not determine current HIV status. In this analysis, HIV and syphilis diagnoses were based on laboratory testing conducted at trial enrolment. The primary outcome was the spatial distribution of syphilis prevalence by administrative division.

### Spatial analyses

This present analysis utilized de-identified data from the STOP trial, accessed on 2nd August 2022. We explored two spatial autocorrelation methods: (1) Global Moran's I test to measure the overall spatial clustering of syphilis prevalence rates across divisions; (2) Local Indicators of Spatial Association (LISA) to identify and map specific divisions where syphilis prevalence was significantly clustered, highlighting potential hotspots or coldspots (15). Neighbouring divisions were determined using the first-order queen polygon continuity method (16). We used a Kulldorff Spatial-Scan Poisson model (SaTScan) to detect divisions with high or low syphilis prevalence while adjusting for the underlying population at risk in Kampala and Wakiso districts. Because sub-division population estimates specific to pregnant women aged 18–35 years were unavailable in the National Census report*,* the population-at-risk denominator was estimated using census data on young women aged 15–34 years who were currently or had been in a recent marital relationship within the 12 months preceding enrolment within the respective divisions (17). This approach aligns with nationally reported demographic age bands used in census and population-based surveys and has been applied in prior spatial analyses of HIV and sexually transmitted infections in Uganda and similar sub-Saharan African settings (9, 18, 19).

The number of persons diagnosed with syphilis in a division was assumed to be Poisson-distributed according to an underlying population, young women at risk.

Based on the Uganda Population-based HIV Impact Assessment survey, the baseline risk of having multiple sexual partners in the past 12 months was assumed to be 19% for all young women and 14% for those living with HIV (6). We computed the spatial scan statistic using the Kulldorff Spatial Scan Poisson model. Statistical significance was determined using 9,999 Monte Carlo replications (*p* < 0.05). Divisions were considered high-prevalence if they had statistically significant spatial scan statistics, i.e., when the log-likelihood ratio (LLR) exceeded the critical value generated from the Monte Carlo simulations. Therefore, syphilis prevalence clustering referred to statistically significant spatial aggregation of syphilis prevalence at the administrative division level, as identified using spatial autocorrelation and scan statistic methods.

### Regression analysis

Divisions were classified as either high prevalence or low prevalence (HP/LP) based on the spatial scan statistic set at 5% significance, thus creating a binary outcome variable to indicate syphilis clustering. Using modified Poisson regression, we estimated adjusted prevalence ratios for sociodemographic and bio-behavioural HIV risk factors associated with residence in divisions classified as high-syphilis prevalence (HP) vs. low-syphilis prevalence (LP) based on a 5% significance level. Covariates that had potential influence on health-seeking behaviour were included; marital status, history of unintended pregnancy, and HIV testing history (20). Data were analysed using SaTScan and STATA 18. Spatial analysis was conducted using Quantum-GIS (QGIS) and GeoDa.

### Ethics approval

The STOP trial was approved by the Joint Clinical Research Centre Research Ethics Committee (JC1214), the Uganda National Council for Science and Technology (HS1681), and the Johns Hopkins IRB (NA_00012998/CR00015330). All study participants in the STOP trial gave informed written consent for randomization and for temporary specimen storage (blood) (13).

## Results

Of 442 pregnant women with syphilis in the STOP study, 422 were included in the present analysis. The remaining 20 women were excluded because they were either older than 35 years or resided outside the 12 divisions in the study catchment area. The median age was 26 years [interquartile range (IQR): 24–29]. About half (224, 53%) were employed, most (314, 74%) were in monogamous marriages, and half (50%) had attained secondary level education (Table 1). The majority (378, 90%) reported at least two prior pregnancies, 319 (76%) reported ever experiencing a stillbirth, and 125 (30%) reported having ever had an unintended pregnancy. At enrolment, 252 (60%) women reported HIV testing in the past three months, and 26 (6%) were diagnosed with both HIV and syphilis. Eighty-one male partners (19%) were enrolled in the study; the median age was 30 years (IQR 28.0, 39.0), and almost all 78 (97%) were employed. Of these, 23 (28%) reported having other sexual partners, of whom 78% (18/23) reported more than two partners.

**Table 1:** Participant characteristics across the prevalence clusters.

Participant characteristics	General (*N* = 422)	Low prevalence (151, 35.8%)	High prevalence (271, 64.2%)
Age of participant			
Median (Q1, Q3)	26.0 (24.0, 29.0)	26.0 (24.0, 29.0)	26.0 (24.0, 29.0)
Education level			
Primary	178 (42.2%)	57 (32.0%)	121 (68.0%)
Secondary	211 (50.0%)	81 (38.4%)	130 (61.6%)
Tertiary	33 (7.8%)	13 (39.4%)	20 (60.6%)
Marital status^a^			
Married monogamous	314 (74.4%)	103 (32.8%)	211 (67.2%)
Married polygamous	90 (21.3%)	44 (48.9%)	46 (51.1%)
Cohabiting	18 (4.3%)	4 (22.2%)	14 (77.8%)
Undertaken paid work prior 3 months			
No	194 (46.0%)	74 (38.1%)	120 (61.9%)
Yes	228 (54.0%)	77 (33.8%)	151 (66.2%)
Main employment status			
Unemployed/Not working	198 (46.9%)	74 (37.4%)	124 (62.6%)
Self-employed/Small business	192 (45.5%)	64 (33.3%)	128 (66.7%)
Public/Private employee	32 (7.6%)	13 (40.6%)	19 (59.4%)
Ever had an unintended pregnancy^a^			
No	297 (70.4%)	91 (30.6%)	206 (69.4%)
Yes	125 (29.6%)	60 (48.0%)	65 (52.0%)
Prior pregnancy outcome (self-reported)^a^			
Still births (fetal loss)	319 (75.6%)	101 (31.7%)	218 (68.3%)
Miscarriage	60 (14.2%)	27 (45.0%)	33 (55.0%)
Others	43 (10.2%)	23 (53.5%)	20 (46.5%)
Presence of genital ulcers	9 (2.1%)	4 (44.4%)	5 (55.6%)
Gravid categories			
1	44 (10.4%)	16 (36.4%)	28 (63.6%)
2+	378 (89.6%)	135 (35.7%)	243 (64.3%)
HIV testing history^a^			
≤3 months	170 (40.3%)	44 (25.9%)	126 (74.1%)
>3 months	252 (59.7%)	107 (42.5%)	145 (57.5%)
HIV status^b^			
Positive	26 (6.2%)	14 (53.8%)	12 (46.2%)
Negative	396 (93.8%)	137 (34.6%)	259 (65.4%)
Male Partners			
Age of Partner			
Median (Q1, Q3)	30.0 (28.0, 39.0)	31.0 (28.0, 37.0)	30.0 (27.0, 40.0)
Partner employed	78 (97.5%)	30 (38.5%)	48 (61.5%)
Partner's years of work			
Median (Q1, Q3)	8.0 (4.0, 15.0)	8.0 (3.0, 10.0)	9.0 (4.0, 15.0)
Having ≥ 1 sexual partner	23 (29.1%)	7 (30.4%)	16 (69.6%)
Sexual Partners^c^			
1 partner	5 (21.7%)	0 (0.0%)	5 (100%)
≥2 partners	18 (78.3%)	7 (38.9%)	11 (61.1%)
Partner condom use with			
No	16 (59.3%)	7 (43.8%)	9 (56.2%)
Yes	11 (40.7%)	3 (27.3%)	8 (72.7%)
Partner syphilis results			
Negative	58 (75.3%)	22 (37.9%)	36 (62.1%)
Positive	19 (24.7%)	8 (42.1%)	11 (57.9%)

a,b,c Statistically significant at *p* < 0.01, *p* < 0.05 and *p* < 0.2 respectively.

Across the 12 divisions where women with syphilis resided, prevalence varied. Highest prevalence was observed in Kawempe division (30.6%), followed by Kasangati Town Council (19.2%) and Rubaga division (13.0%) while lowest in Kajjansi Town Council (2.1%), Gombe division (1.9%) and Kyengera Town Council (1.4%) (Table 2).

**Table 2:** Local Moran's I test.

Division	Syphilis prevalence	Moran Statistic	*P*-value	LISA classes
Kawempe Division	30.60%	1.06	0.019	High-High
Kasangati Town Council	19.20%	0.51	0.193	High-High
Rubaga Division	13.30%	0.02	0.799	High-High
Nabweru Division	12.90%	0.54	0.045	High-High
Makindye Division	5.10%	0.02	0.898	Low-Low
Ndejje Division	5.10%	0.28	0.255	Low-Low
Wakiso Division	3.70%	0.06	0.819	Low-Low
Central Division	2.30%	−0.64	0.1	Low-High
Kira Division	2.30%	−0.85	0.254	Low-High
Kajjansi Town Council	2.10%	0.42	0.343	Low-High
Gombe Division	1.90%	−0.65	0.236	Low-High
Kyengera Town Council	1.40%	0.21	0.434	Low-Low

Using the first-order queen polygon, each division had neighbouring divisions in the range of 1–5, and the mean number of neighbours was 2.5. Global Moran's I revealed a weak tendency towards syphilis clustering that was not statistically significant (Moran's I = 0.08, *p* = 0.19), suggesting syphilis was randomly distributed across the divisions of Kampala and Wakiso districts. The Local Moran's matrix revealed that most neighbouring divisions had similar prevalence, except for Kawempe division (I = 1.06, *p* = 0.019), which neighboured Nabweru division in the north (I = 0.54, *p* = 0.045) (Table 2).

**Figure 1:**
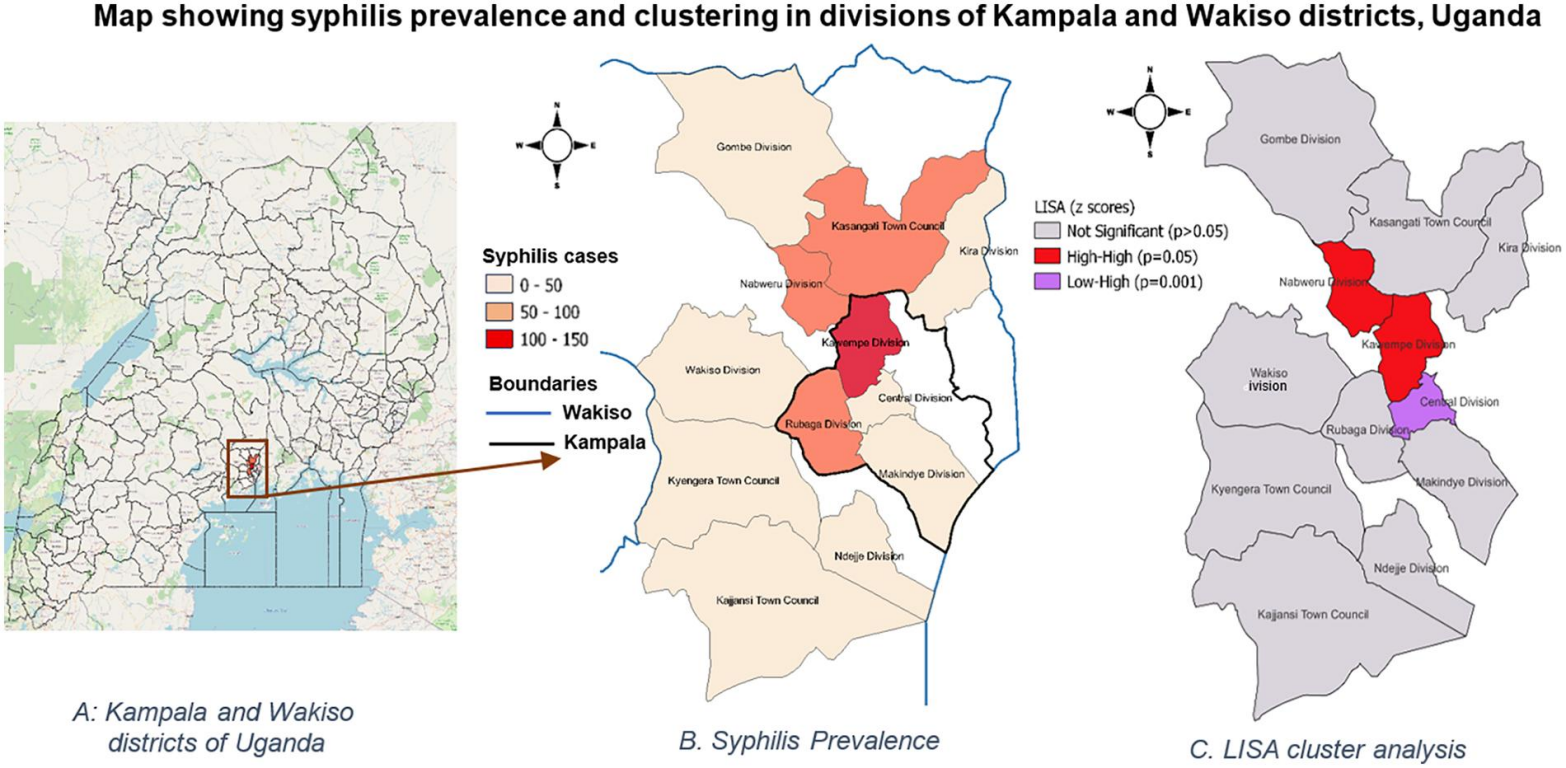
Prevalence and clustering of syphilis in divisions of Kampala and Wakiso districts, Uganda. Shape files of Uganda Administrative boundaries are open source information obtained from the website: “https://data.humdata.org/dataset/cod-ab-uga” which source data from Uganda Bureau of Statistics with support from WHO.

Syphilis prevalence was highest in Kawempe division and its neighbouring divisions: Rubaga in the south, and Nabweru and Kasangati Town Council in the north (Figure 1B**)**. LISA analysis revealed that high syphilis prevalence and neighbouring divisions in the north were statistically related (Kawempe and Nabweru, *p* = 0.05). Furthermore, the low prevalence in the Central division and the high prevalence in Kawempe division in the south were statistically associated (Central and Kawempe divisions, *p* = 0.001) (Figure 1C).

Based on Kulldorff spatial scan (ScanSta) modelling, four divisions had statistically significant rates of syphilis indicated by the highest relative risk, and were classified as High Prevalence (HP) divisions: Kasangati Town Council [Relative Risk (RR) = 2.79, *p* < 0.0001], Kawempe (RR = 2.52, *p* < 0.0001), Nabweru (RR = 1.95, *p* = 0.0002), and Kyengera Town Council (RR = 0.12, *p* < 0.0001) (Table 3). Nine (75%) of the 12 divisions had at least one woman living with HIV; however, HIV clustering was not statistically significant across any of the divisions (*p* > 0.05).

**Table 3:** Kulldorff spatial analysis showing distribution of syphilis cases in statistically significant high prevalence divisions.

Division	Population of young women (18–35 years)	Cases	Expected cases	Log-likelihood ratio	RR	*P*-value
Kasangati Town Council	656	81	33.06	27.7	2.79	<0.0001
Kawempe Division	1,267	131	63.84	33.63	2.52	<0.0001
Nabweru Division	597	55	30.08	9.07	1.95	0.0002
Kyengera Town Council	909	6	45.8	29.62	0.12	<0.0001

Population estimates were derived from the Uganda National Population and Housing Census and represent projected counts of women aged 15–34 years residing in each division (25).

Among all the women enrolled, 271 (64%) were residents of HP divisions. More women in HP divisions had an HIV test compared to LP divisions (*p* < 0.0001), while more women in HP divisions had negative HIV results (*p* = 0.045). Participant age, gravidity, and partner age did not differ significantly between high- and low-prevalence divisions and were not significantly associated with residence in high-prevalence divisions.

Multivariable analysis revealed that syphilis prevalence in HP divisions was negatively associated with being in a polygamous relationship, having prior unintended pregnancy, and having done HIV testing beyond three months prior (Table 4). Women in polygamous relationships were 36% less likely to be living in HP divisions [adjusted prevalence ratio [APR] = 0.64; 95% confidence interval [CI] 0.47, 0.88; *p* = 0.005]. Similarly, women with a history of an unintended pregnancy were 22% less likely to be residents in HP divisions (APR = 0.78, 95% CI: 0.64, 0.93, *p* = 0.007). In comparison, women with long duration (at least three months) since the last HIV test were 17% less likely to live in HP divisions (APR = 0.83, 95% CI: 0.72, 0.95, *P* = 0.008). However, women with negative HIV serostatus were 39% as likely to reside in an HP division, but this was not statistically significant (APR = 1.39, 95% CI: 0.91, 2.11, *p* = 0.12).

**Table 4:** Factors associated with living in a high prevalence division.

Factors	Unadjusted prevalence ratio (95% CI)	*P*-value	Adjusted prevalence ratio (95% CI)	*P*-value
Marital status				
Cohabiting	Ref	–	Ref	–
Married monogamous	0.86 (0.67, 1.12)	0.27	0.82 (0.64, 1.05)	0.12
Married polygamous	0.66 (0.48, 0.90)	0.01	0.64 (0.47, 0.88)	0.005
Ever had an unintended pregnancy				
No	Ref	–	Ref	–
Yes	0.75 (0.62, 0.90)	0.002	0.78 (0.64, 0.93)	0.007
HIV testing history				
≤3 months	Ref	–	Ref	–
>3 months	0.78 (0.68, 0.89)	<0.001	0.83 (0.72, 0.95)	0.008
HIV status				
Positive	Ref	–	Ref	–
Negative	1.42 (0.93, 2.16)	0.11	1.39 (0.91, 2.11)	0.12

## Discussion

This study focused on new insights into the spatial epidemiology of syphilis among young pregnant women aged 18–35 years in Kampala and Wakiso districts. We found that syphilis was clustered in divisions with high syphilis prevalence, and divisions neighbouring those with high prevalence. We identified significant clustering of syphilis cases in specific urban and peri-urban divisions, notably Kasangati Town Council, Kawempe, and Nabweru. Syphilis prevalence clustering was negatively associated with residence in high-prevalence divisions among women in polygamous relationships, those with prior unintended pregnancy, and had tested for HIV more than three months prior. These patterns likely represent differences in health seeking behaviour, service access, and intensity of HIV and/or syphilis case detection rates across divisions, rather than causal protective effects. Spatial analysis adds value in its ability to identify high-burden areas for targeted intervention. While national policies advocate for universal screening (7), our results suggest that intensified efforts such as enhanced partner notification, community outreach, and resource allocation may be warranted in identified hotspots. Therefore, this approach can improve the efficiency and effectiveness of syphilis elimination programs, particularly in resource-limited settings like Uganda.

We identified high syphilis prevalence among young women residing in Kawempe, Nabweru, and Rubaga divisions and Kasangati Town Council. Across all divisions, global Moran's I detected random patterns of syphilis prevalence. However, from the individual analysis of Local Moran's I, clustering was detected within certain hotspots, such as Kawempe and Nabweru divisions. Furthermore, LISA analysis revealed spatial clustering in certain divisions, notably Nabweru and Kawempe, where the prevalence was significantly higher (high-high). Bwaise, one of Kampala's biggest slums, is located in Kawempe division and has an HIV prevalence of 25.4% (five times the national prevalence) due to transactional sex, drug/alcohol use, poverty, and inconsistent condom use in bar settings (21). Both Kawempe and Nabweru divisions have large populations, an informal economy, and brothels, factors found in previous studies across sub-Saharan Africa to influence sexual behaviour and STI incidence (22, 23).

On the other hand, the Central Division of Kampala District had a lower syphilis prevalence despite being surrounded by higher-prevalence divisions. This could be because the Central Division is primarily commercial, has the smallest population among Kampala city divisions, and has a high concentration of health facilities, thereby enabling better access to health services. A previous study in South Africa highlighted a similar pattern in clusters of STI prevalence; areas with better access to preventive services had significantly lower STI prevalence than their neighbouring districts (24). Understanding these protective factors could inform strategies for controlling syphilis in neighbouring high-prevalence areas. Overall, spatial analysis of syphilis prevalence detailed the importance of geographically targeted interventions, as highlighted in similar STI research in South Africa (24). In our analyses, one-third were highly prevalent (HP) divisions, with the number of syphilis cases almost twice that of low-prevalent (LP) divisions, which was evidence for pockets of syphilis prevalence. The risk of syphilis was highest in Kasangati Town Council, followed by Kawempe and Nabweru divisions, and Kyengera Town Council. These divisions share several socio-demographic, geographic, and infrastructural characteristics that would explain the clustering of STIs like syphilis. They are peri-urban or urbanizing areas characterized by high population density, large informal settlements, and limited access to consistent, high-quality healthcare (25). A systematic review of 23 studies in sub-Saharan Africa revealed that peri-urban areas often serve as hotspots for STIs due to rapid urbanization, weak health systems, and socio-economic vulnerability (26). HP divisions in our study had dense populations with high levels of informal economic activity and a young demographic, factors associated with increased sexual risk behaviours and limited access to youth-friendly sexual and reproductive health services (25, 27). They are located along key transport routes, where higher mobility, economic insecurity, and transactional sex are commonly reported—conditions shown to drive STI transmission in urban communities (28, 29).

We observed similar proportions of women in polygamous relationships across HP and LP divisions. Women residing in HP divisions were more likely to have had an HIV test in the prior three months and were aware of their HIV status. Conversely, women who were in polygamous relations, or those with history of unintended pregnancy, or whose last HIV test over three months before the study, were less likely to reside in HP divisions. Such inverse relationships with place of residence could reflect differences in health-seeking behaviour, service availability, and levels of testing and case detection of infections across divisions. Previous studies have documented similar patterns showing that in more stable, low-transmission communities, polygamous relationships may coexist with structured social norms and established pathways to care, potentially fostering patterns of service utilization rather than biological risk (30, 31). Similarly, women who had experienced unintended pregnancies may have interacted more frequently with maternal and reproductive health services which could influence both detection and reporting of sexually transmitted infections (32, 33). In addition, the finding that women who had last tested for HIV more than three months prior were more likely to be in LP divisions may reflect a lower perceived or actual risk of recent exposure, but may also indicate reduced testing frequency or lower coverage of HIV and syphilis screening services in these divisions.

In Uganda, the social context in which young women are embedded may also play a role in influencing their behaviours and health outcomes. By contrast, socially and economically disadvantaged communities may offer few sexual reproductive resources to help young women develop physical, social, and emotional competencies necessary for reaching their full potential for health and well-being. Therefore, if real-level contextual factors contribute to sexual behaviour, thus influencing the syphilis prevalence, then differential prevalence should be expected within large geographical areas such as divisions in which variations in these same factors are present.

Therefore, our findings reveal factors associated with clustering; women in polygamous relationships, those with a history of unintended pregnancy, and those who had not recently tested for HIV were less likely to reside in high-prevalence divisions. These associations may reflect differences in health-seeking behaviour, access to services, or underlying social determinants. However, none of these factors should detract from the need for universal syphilis screening and treatment in pregnancy, as recommended by WHO and national guidelines.

This study had several strengths. First, the application of spatial epidemiological methods—including LISA and Kulldorff's spatial scan statistics—enabled precise identification of syphilis hotspots, providing granular, location-specific insights that are critical for targeted interventions. Such methodological approaches align with global best practices in STI surveillance and support the use of geospatial intelligence in public health decision-making (34, 35). The study focused on urban and peri-urban areas within the Kampala-Wakiso corridors, thereby offering a valuable contribution to understanding intra-urban inequalities in sexual health outcomes —a growing area of interest in rapidly urbanizing low- and middle-income countries. Integrating individual-level behavioural and socio-demographic data with area-level clustering further enriched the analysis, allowing for a nuanced exploration of the interplay between personal risk factors and spatial vulnerability.

Our analysis had limitations. First, the available data were obtained from a single site (Mulago Hospital). However, the site had a large catchment area, which presents a potential bias in the data that may affect generalizability. Second, use of census-derived proxy denominators rather than pregnancy-specific population counts may have introduced imprecision in relative risk estimates. However, similar approaches are widely used in subnational spatial epidemiology when risk-specific denominators are unavailable and have been shown to provide useful insights for public health planning (9, 18, 19). The available data did not include sexual risk behaviour and alcohol use, yet they may influence or confound the associations with syphilis/HIV clustering. Additionally, the relatively low mobility of pregnant women may limit the interpretation of spatial clustering, as they receive care from a single facility until the due date, compared to studies of the general population. Finally, the proportion of young women at risk of syphilis/HIV infection was unknown because general population surveys did not disaggregate data for pregnant young women. We assumed the proportion of the young women's population at greatest risk, as we wanted to be conservative in our estimation.

## Conclusions

In conclusion, our analyses support the integration of spatial analysis into routine surveillance and program planning for syphilis elimination. By the approach of identifying syphilis hotspots, especially among pregnant women, we can develop more precise targeted interventions and equitable allocation of resources across regions and subnational units.

While universal antenatal syphilis screening remains the cornerstone of congenital syphilis prevention, spatial identification of high-prevalence divisions can complement these efforts by enabling more targeted interventions, including enhanced outreach, partner notification, and resource allocation in high-burden areas of Uganda. Additionally, targeted outreach should prioritize pregnant women who present with a history of undesired pregnancy, being in a polygamous relationship, and having had an HIV test beyond 3 months. Therefore, tailoring STI/HIV programs that address both geographic and specific individual-level characteristics or target populations will help reduce the burden of congenital syphilis and advance maternal and child health outcomes in Uganda.

## Data Availability

The dataset is publicly available on the repository with this DOI: https://doi.org/10.5281/zenodo.16874894.
